# Past, Current, and Future of Immunotherapies for Prostate Cancer

**DOI:** 10.3389/fonc.2019.00884

**Published:** 2019-09-11

**Authors:** Adeline N. Boettcher, Ahmed Usman, Alicia Morgans, David J. VanderWeele, Jeffrey Sosman, Jennifer D. Wu

**Affiliations:** ^1^Department of Urology, Feinberg School of Medicine, Northwestern University, Chicago, IL, United States; ^2^Department of Hematology and Oncology, Feinberg School of Medicine, Northwestern University, Chicago, IL, United States; ^3^Department of Microbiology and Immunology, Feinberg School of Medicine, Northwestern University, Chicago, IL, United States

**Keywords:** prostate cancer, metastatic-castration resistant prostate cancer, immunotherapy, combination therapy, immune checkpoint inhibitor

## Abstract

Prostate cancer (PCa) is the most common cancer in men, and the second leading cause of cancer related death in men in Western countries. The standard therapy for metastatic PCa is androgen suppression therapy (AST). Men undergoing AST eventually develop metastatic castration-resistant prostate cancer (mCRPC), of which there are limited treatment options available. Immunotherapy has presented substantial benefits for many types of cancer, but only a marginal benefit for mCRPC, at least in part, due to the immunosuppressive tumor microenvironment (TME). Current clinical trials are investigating monotherapies or combination therapies involving adoptive cellular therapy, viral, DNA vaccines, oncolytic viruses, and immune checkpoint inhibitors (ICI). Immunotherapies are also being combined with chemotherapy, radiation, and AST. Additionally, preclinical investigations show promise with the recent description of alternative ways to circumvent the immunosuppressive nature of the prostate tumor microenvironment, including harnessing the immune stimulatory NKG2D pathway, inhibiting myeloid derived suppressor cells, and utilizing immunomodulatory oncolytic viruses. Herein we provide an overview of recent preclinical and clinical developments in cancer immunotherapies and discuss the perspectives for future immunotherapies in PCa.

## Introduction

Prostate cancer (PCa) is the most commonly diagnosed malignancy and ranked the second leading cause of cancer related death in US men (1). Radical prostatectomy and/or radiation are the standard primary treatments for patients with localized PCa, while recurrent disease and/or advanced staged PCa, the main therapy is androgen ablation (2) with or without therapy intensification. Despite initial effective responses with androgen suppression therapy (AST), almost all patients ultimately progress to metastatic castration-resistant prostate cancer (mCRPC) (3). Docetaxel, abiraterone, enzalutamide, cabazitaxel, and Sipuleucel-T (Sip-T) (4–9) are approved by the FDA for the treatment of mCRPC, however each of these regimens provides only limited 2–4 months median survival benefit. The median overall survival (OS) for mCRPC patients ranges from 13–32 months with a 15% 5-year survival rate (10, 11). Therefore, efforts to explore new therapeutic modalities for mCRPC are urgently needed.

In the last decade there have been significant milestones achieved for immunotherapies. In 2010, the FDA approved the first dendritic cell based vaccine, Sip-T for the treatment of non-symptomatic metastatic prostate cancer (9). Following this approval, the immune checkpoint CTLA-4 inhibitor, ipilimumab, was approved for the treatment of metastatic melanoma in 2011 (12). Shortly thereafter, immune checkpoint PD-1/PD-1L inhibitors were approved starting in 2014 for a variety of cancers including lung cancer, kidney cancer, urothelial cancer, Hodgkin's disease, breast cancer (13), as well as for microsatellite high and mismatch repair deficient solid tumors (14). Other than Sip-T, no other immunotherapeutic modality is approved for use in PCa. However, there are currently many ongoing immunotherapeutic clinical trials to assess their immune and clinical efficacy. In the present review, we will discuss advances made in preclinical trials and the tools used in this research and recent clinical trials.

## Ongoing Clinical Trials

**Figure 1:**
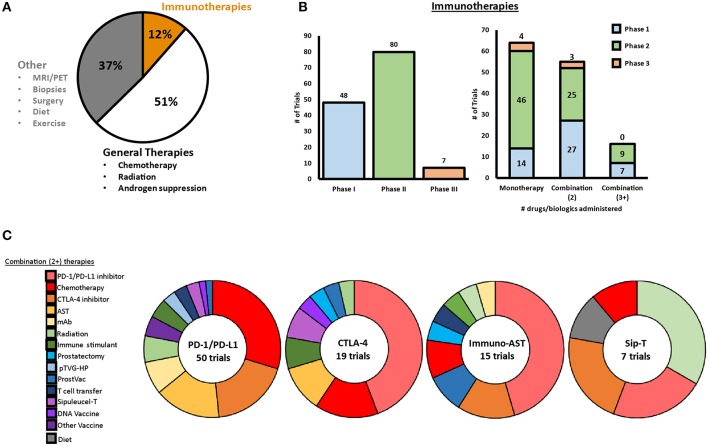
Snapshot of clinical trials ongoing for prostate cancer in 2019. **(A)** As of May 2019, a total of 1,195 clinical trials were ongoing for prostate cancer, with 12% of these trials involving at least one immunotherapeutic. **(B)** Of the ongoing immunotherapeutic trials, two are in early Phase, 46 are in Phase I, 84 are in Phase II, and seven are in Phase III. Approximately half of the ongoing trials are testing combination therapies with two or more therapeutics. **(C)** PD-1/PD-L1 blockade, CLTA-4 blockade, AST, and Sip-T are implemented in combination clinical trials with a variety of different therapies.

Several clinical trials have been completed or are ongoing to investigate diverse immunotherapeutic approaches for patients with mCRPC, including vaccines, immune checkpoint inhibitors (ICI) and immunomodulators, adoptive cell transfer (ACT), oncolytic virus-mediated immune response, as well as combinatorial approaches with radiation, chemotherapy, and others. As of May 1, 2019, more than 1100 clinical trials are active (or recruiting) for PCa spanning these different approaches, of which 63% are therapy based, with 12% being immunotherapeutic in nature. Figure 1 shows a snapshot of ongoing clinical trials for prostate cancer in mid-2019, with a specific focus on immunotherapies that are being investigated. We provide a detailed description of each type of immunotherapy, as well as provide an overview of ongoing clinical trials for each type of therapy throughout this review.

### Vaccine Based Therapies

#### DNA-Based Vaccines

DNA vaccines elicit an immune response by being transfected, transcribed, and translated by host cells to produce foreign antigens that are recognized by the immune system. DNA vaccines can be designed to exploit tumor associated antigens (TAAs) to mount an immune response that produces a robust proliferation and activation of tumor antigen specific T cells (15–18), and are an area of current investigation. PCa has a variety of TAAs that can be targeted, including prostate-specific antigen (PSA), prostate-specific membrane antigen (PSMA), prostatic acid phosphatase (PAP), prostate stem cell antigen (PSCA), prostein, T cell receptor gamma alternate reading frame protein (TARP), Trp-p8, and Six-transmembrane epithelial antigen of the prostate 1 (STEAP1), and NY-ESO-1 (19, 20).

One of the most studied prostate cancer DNA vaccines encodes PAP plus granulocyte-macrophage colony stimulating factor (GM-CSF) (pTVG-HP), which has been tested in a phase I trial of men with biochemically recurrent PCa. Patients in this trial were treated six times every 2 weeks with pTVG-HP and GM-CSF. Results from the phase I trial show that the vaccination is safe and that 9 of 22 patients developed CD4^+^ and/or CD8^+^ PAP specific responses, and patient PSA doubling time increased from 6.5 months pre-treatment to 9.3 months 1 year post treatment (21). In a follow up study by the same group, patient PBMC samples from the original phase I cohort were further assessed for antigen specific T cell populations and responses. This study found that multiple immunizations were necessary for robust responses as a majority of the PAP specific T cells were not detectable until after the 6 vaccination regimen was completed (22). pTVG-HP is currently being combined with Sip-T (NCT01706458) and nivolumab (NCT03600350) in phase II clinical trials.

Neoantigen DNA vaccinations are being developed as a personalized therapeutic to stimulate T cells to specifically attack neoantigen bearing tumor cells. Neoantigen DNA vaccines are developed through the identification of tumor specific antigens through whole exome sequencing of tumor and germline DNA (23). Neoantigen DNA vaccinations are still in early stages of development, however dendritic cell (24) and peptide based (25, 26) neoantigen vaccinations have been successful in eliciting T cell responses in preliminary clinical studies performed in patients with melanoma, primary Glioblastoma, and lung cancer, suggesting that neoantigen DNA vaccinations have the potential to be efficacious in PCa as well. An intensive phase I trial has been initiated that involves a combination treatment regimen with a neoantigen DNA vaccination, nivolumab, ipilimumab, and PROSTVAC in patients with metastatic hormone sensitive prostate cancer (NCT03532217).

#### Cell-Based Vaccines

Cell-based vaccination generally consists of autologous or allogenic whole cells, including antigen presenting cells (APCs) and PCa cells that are modified to bear TAAs and/or GM-CSF to induce anti-tumor immune responses (27). One cell-based vaccination under investigation is GVAX, which is a whole cell vaccine comprised of castration-sensitive and castration-resistant allogenic prostate cancer cell lines (LNCaP and PC3, respectively). The cell lines are engineered to overexpress GM-CSF, resulting in the activation of DCs, and then subsequently T cells, to elicit robust antitumor responses. In an early phase I/II trial, patient survival was extended to 24 months in the low dose cohort (100 million cell booster) and 36.9 months in the high dose cohort (300 million cell booster) compared to a predicted 19.5 months (27). Although survival was extended in early trials, two phase III studies (VITAL-1 and VITAL-2) failed to show improved outcomes and were closed early due to the lack of clinical efficacy (28, 29). A similar phase I trial was performed in which patient tumors were resected and then underwent retroviral transductions to express GM-CSF; vaccination activated new T and B cell responses against PCa antigen (30). GM-CSF cellular vaccinations are currently not being tested in the clinics for PCa. However, GM-CSF is still being investigated for its use in other types of PCa vaccines in preclinical testing, such as in combination with norcantharidin (31).

Sip-T is an autologous dendritic cell vaccine generated by *ex vivo* priming of patient DCs with PA2024 (fusion protein with PAP and GM-CSF) (32, 33). Sip-T was the first FDA approved therapeutic cancer vaccine in 2010. Three multicenter phase III clinical trials were performed to assess the efficacy in asymptomatic or minimally symptomatic patients with mCRPC. The first two trials showed no difference in time to tumor progression (TTP), but demonstrated a statistically improved OS benefit among patients treated with Sip-T [25.9 vs. 21.4 months (*P* = 0.01, HR, 1.7), and 19.0 vs. 15.7 months (*P* = 0.3, HR, 1.27)] (34, 35). A third Phase III clinical trial (IMPACT) enrolled 512 patients who were randomly assigned in a 2:1 ratio to Sip-T or placebo. Similarly to the previous two trials, patients receiving Sip-T had a median 4.1 month OS benefit compared to the placebo [25.8 vs. 21.7 months (*P* = 0.02, HR, 0.77)] while having no significant difference in TTP (14.6 vs. 14.4 weeks) (9). Safety data demonstrated that treatment was overall well-tolerated with no severe adverse events (36). Despite its efficacy and safety, Sip-T is not widely accepted, mainly due to the high cost compared to the degree of benefit (37). Together, these studies ultimately led to the approval of Sip-T for mCRPC. Combination treatments are being investigated in the clinic to enhance the efficacy of Sip-T and include combination with Atezolizumab (Anti-PD-L1) (NCT03024216), Ipilimumab (Anti-CTLA-4) (NCT01804465), radiation (NCT02463799, NCT01818986, NCT01807065), and chemotherapy (NCT01420965).

Chimeric antigen receptor (CAR) T cells are autologous cells that are engineered *ex vivo* to express a TCR signaling domain fused with variable regions of an antibody, enabling them to recognize tumor surface antigens in an MHC independent manner (38). CAR T cells targeting CD19 have shown complete responses in B-cell hematologic malignancies (39), suggesting a promising approach with CAR T cells for treating tumors. A preclinical model utilizing a 4-1BB containing CAR showed potent anti-tumor activity in an LAPC-9 xenograft model (40). Currently clinical trials involving CAR T cells targeting EpCAM (NCT03013712), PSCA (NCT02744287), PSMA (NCT01140373, NCT03089203), and NY-ESO-1 (NCT03159585) are ongoing.

#### Peptide-Based Vaccines

Personalized peptide vaccinations (PPV) utilize immunization with tumor specific peptides that can elicit an immune response to induce cytotoxic T lymphocyte (CTL) activation and subsequent anti-tumor responses. The standard procedure for determining peptide candidates for vaccination is to screen pre-vaccination patient peptides for their ability to induce a CTL or humoral responses to the peptides *in vitro* (41). Targets have been identified for HLA-A24^+^ PCa patients, including PAP (42), PSA (43), and PSMA (44).

A randomized phase II study testing the combination treatment of a PPV and estramustine phosphate (EMP) or EMP alone showed improved PFS (8.5 vs. 2.8 months) for the combination treatment, and was deemed tolerable and safe for ongoing future clinical trials (45). Another randomized phase II trial reported that the OS of docetaxel-resistant CRPC patients showed improved OS to patients receiving PPV compared to those who did not (17.8 vs. 10.5 months) (46). Based on these findings, a phase III, randomized, placebo-controlled trial testing PPV in docetaxel-refractory mCRPC patients is ongoing (UMIN000011308).

A phase I/IIa dose escalation trial with a peptide vaccine UV1, containing a peptide fragment from telomerase reverse transcriptase (hTERT), was performed with patients who had metastatic hormone-naïve prostate cancer. Overall, a majority of the patients responded to therapy as immune responses were detected in 18 of 21, PSA levels declined in 14 of 21, and 10 of 21 had no evidence of persisting tumors by MRI imaging (47). This phase I/IIa trial is still ongoing (NCT01784913), and there are currently no ongoing phase III trials for testing the efficacy of UV1 for PCa.

#### Viral Vector-Based Vaccines

Viral-based vaccines are an immunotherapeutic strategy that utilizes recombinant viral vectors carrying gene sequences of TAAs to mimic natural infection of host immune cells causing specific immune responses against encoded tumor antigens (48). PROSTVAC (TRICOM) is a poxviral-based vaccine regimen consisting of a recombinant attenuated vaccinia and fowlpox virus booster engineered to encode TAAs (PSA) and three costimulatory proteins: B7-1 (CD80), lymphocyte function-associated antigen 3 (LFA-3) (CD58), and intercellular adhesion molecule-1 (ICAM-1) (CD54) (49). A phase II trial of 125 patients with minimally symptomatic mCRPC randomized patients to receive PROSTVAC or a placebo in a ratio of 2:1. Median OS for patients given PROSTVAC was increased over placebo [25.1 vs. 16.6 months (*P* = 0.0061, HR, 0.56)], but not for progression free survival (PFS) (50). Another phase II clinical trial was performed with PROSTVAC and GM-CSF in 32 men with mCRPC, which showed similar findings with an increased median OS of 26.6 months (predicted 17.4 months by Halabi nomogram) (51). These data led to a randomized, placebo-controlled phase III trial in men with mCRPC. Gulley et al. (52) recently published results from this trial; patients were randomized into three groups that received either PROSTVAC, PROSTVAC + GM-CSF, or placebo. Neither treatment influenced median OS (34.4, 33.2, and 34.2 months, respectively), and patients alive without events (AWE) were similar at 6 months (29.4, 28.0, and 30.3%, respectively). Overall, PROSTVAC was safe, but it did not affect OS or AWE in patients with mCRPC (52). These new results suggest that PROSTVAC as a single agent is not effective, and that an immunosuppressive environment could negatively influence the efficacy of the treatment. Combination therapies with PROSTVAC are being investigated for their ability to enhance immune responses. Ongoing combination clinical trials with PROSTVAC include either DNA vaccines (NCT03532217), ICI (NCT02506114, NCT02933255), and chemotherapy (NCT02649855).

Adenovirus 5 (Ad5) is another important vector that is used as an immune agent to immunize against TAAs. A phase I trial was performed to study the immune responses to an Ad5-PSA vaccination in 32 patients with hormone refractory metastatic prostate cancer. In response to vaccination, 34% of patients generated anti-PSA antibodies, 68% developed anti-PSA T cell responses, 48% had an increased PSA doubling time, and 55% survived longer than predicted (53). An ongoing Phase II clinical trial (NCT00583024) investigating adenovirus/PSA responses in men with hormone refractory prostate cancer showed anti-PSA T cell responses were present in 100% of patients with recurrent disease and 67% in patients with hormone refractory disease (54). A newly recruiting phase I clinical trial will examine how mCRPC patients will respond to adenoviral PSA (ETBX-071), MUC1 (ETBX-061), and ETBX-051 (brachyury) vaccines (NCT03481816). An active Phase I trial is testing ETBX-051, ETBX-061, in addition to an adenoviral CEA vaccine (ETBX-011) (NCT03384316).

### Immune Checkpoint Inhibitors

Immune checkpoint inhibitors (ICIs) are antibodies that target regulatory or co-inhibitory signaling molecules, including cytotoxic T-lymphocyte antigen-4 (CTLA-4), programmed cell death-1 (PD-1), and programmed cell death ligand-1 (PD-L1). Blockade of these inhibitory entities results in subsequent T cell activation and anti-tumor activity (55). To date, PD-1/PD-L1 blockade antibody therapies have been approved for a spectrum of solid tumor malignancies (Table 1). Anti-CTLA-4 antibody therapy is only approved for melanoma and renal cell carcinoma; however, there are multiple ongoing clinical trials with anti-CTLA-4 as one of the combination agents. No ICIs are approved specifically for the treatment of PCa, other than for tumors with high microsatellite instability, due to their marginal efficacy in clinical trials. Substantial efforts are being made to assess the efficacy of ICI therapies in combination settings for PCa.

**Table 1:** FDA-approved immune checkpoint blocking antibodies.

**Target**	**Antibody drug**	**Trade name**	**Tumor Type (FDA Approval Year)**
PD1	Nivolumab (IgG4)	Opdivo	Melanoma (2014)
			Non-small-cell lung cancer (2015)
			Hodgkin lymphoma (2016)
			Head and neck squamous cell carcinoma (2016)
			Urothelial carcinoma (2017)
			Hepatocellular carcinoma (2017)
	Pembrolizumumab (IgG4)	Keytruda	Melanoma (2014)
			Non-small-cell lung cancer (2015)
			Head and neck squamous cell carcinoma (2016)
			Hodgkin lymphoma (2017)
			Urothelial carcinoma (2017)
			Gastic and gastroesophageal carcinoma (2017)
	Cemiplimab (IgG4)	Libtayo	Cutaneous squamous cell carcinoma (2018)
PD-L1	Atezolizumab (IgG1)	Tecentriq	Urothelial carcinoma (2016)
			Non-small-cell lung cancer (2016)
	Durvalumab (IgG1)	Imfinzi	Urothelial carcinoma (2017)
			Non-small-cell lung cancer (2018)
	Avelumab (IgG1)	Bavencio	Merkel cell carcinoma (2017)
			Urothelial carcinoma (2017)
CTLA-4	Ipilimumab (IgG1)	Yervoy	Melanoma (2014)

#### CTLA-4 Blockade

Ipilimumab, the only FDA approved anti-CTLA-4 monoclonal antibody for treatment of melanoma was and is still being evaluated in several phase I, II, and III clinical trials for patients with PCa as a single agent or combination therapy. In a phase I/II clinical trial for mCRPC, it was shown that ipilimumab was safe, tolerable, and synergistic with radiotherapy. Of 50 patients given 10 mg/kg ipilimumab and radiotherapy, eight had PSA declines (≥50%), one had a complete response, and six had stable disease ranging from 2.8 to 6.1 months (56). A randomized phase III trial was performed to compare ipilimumab with placebo after bone directed radiotherapy in 799 patients who had progressed on docetaxel chemotherapy. In this trial, there was no significant difference in OS between ipilimumab and placebo [11.2 vs. 10 months (*P* = 0.053, H, 0.85)], but a modest benefit in PFS [4.0 vs. 3.1 months (*P* < 0.0001, HR, 0.70)] (57). An unplanned subgroup analysis demonstrated that patients with no visceral metastases, low bone alkaline phosphatase levels of <1.5 times the upper limit of normal and higher hemoglobin (>100 g/L) were more likely to benefit from ipilimumab therapy in terms of overall survival (57). In another phase III trial, ipilimumab was compared with placebo, and there was no significant difference in median OS [28.7 vs. 29.7 months (*P* = 0.3667, HR 1.11)], but slight PFS benefit [5.6 vs. 3.8 months (HR, 0.67)] and a higher PSA response (23% vs. 8%) (58). Due to the ineffectiveness of Ipilimumab alone, it is being tested in a variety of combination trials for patients with PCa, including with radiation (NCT03477864), nivolumab [NCT03333616, NCT03061539, NCT02985957 (Checkmate 650)], chemotherapy (NCT03098160, NCT01688492), and AST (NCT01498978).

#### PD-1/PD-L1 Blockade

PD-1 and PD-L1 inhibitors disrupt the interaction of PD-1 expressed on the T cell surface and PD-L1 expressed on tumor or myeloid cells within the tumor microenvironment. Engagement of the PD-1 receptor induces T cell anergy, and thus PD-1/PD-L1 blockade revives T cell anti-tumor responses. Within the last 5 years, PD-1 and PD-L1 inhibitors have been approved for use in melanoma, non-small-cell cancer, head, and neck squamous cell carcinoma, Hodgkin's lymphoma, urothelial carcinoma, renal cell carcinoma, as well as gastric and gastroesophageal carcinoma [reviewed in (59) and Table 1]. In a phase I trial for mCRPC, the anti-PD1 mAb nivolumab was administered to 17 patients with mCRPC; no objective responses were seen in any patients (60) (NCT00730639). One promising Phase II study recruited 10 patients that previously received enzalutamide and were then treated with pembrolizumab. Three of the patients responded with measurable serum PSA declines, with two of the patients having tumor size reduction in a 12 or 24-week period (61). In another phase Ib trial, 23 mCRPC patients with PD-L1 positive tumors were treated with the anti-PD1 mAb pembrolizumab; 17.4% of patients had an objective response rate, with stable disease in 34.8% of patients, and tumor size reduction in 10 of 21 assessible patients (62). A phase Ia study with atezolizumab, an anti-PD-L1 mAb, in patients with mCRPC showed that the drug was well-tolerated with a 56% 12-month OS (63) (NCT01375842). Together, these results suggest that monotherapy is not sufficient for significant responses in PCa, and thus combinatorial approaches are being investigated. Currently, ongoing PD-1/PD-L1 blockade clinical trials include combination with PROSTVAC (NCT02933255), pTVG-HP (NCT03600350), chemotherapy (NCT03572478, NCT03170960, NCT03673787), radium-223 (NCT03093428, NCT02814669), Sip-T (NCT03024216), and CTLA-4 checkpoint inhibitors (NCT03333616).

#### B7-H3 Blockade

B7-H3 is a newly investigated immune checkpoint target for immunotherapy. B7-H3 shares 20–27% amino acid identity with other B7 family members and is found to be expressed by tumor cells and immature dendritic cells (64). The receptor for B7-H3 is unknown. However, overexpression of B7-H3 on cancer cells inhibits T cell function, and thus contributes to immune evasion (65, 66). Recent studies show that prostate tumors that express B7-H3 are positively correlated with a high Gleason score, mCRPC, and tumor stage (67). Monoclonal antibodies targeting B7-H3 are being tested in the clinic as a monotherapy [NCT02628535, NCT02923180 (68), and NCT01391143] in phase I and II trials.

### Oncolytic Viruses-Mediated Immune Modulation

Another field of viral based cancer vaccines are oncolytic viruses (69–72). Oncolytic viruses are designed to specifically target, replicate in, and kill cancerous cells, while keeping normal cells intact. The field of utilizing oncolytic viruses as an immunotherapeutic for prostate cancer is newly emerging and many studies are still in the preclinical stage to study the effects on the immune system. However, one immunotherapeutic oncolytic virus, Ad5-yCD/mutTKSR39rep-hIL12, is currently in clinical trials. A series of Ad5-CD/TK oncolytic viruses have been developed and tested as a therapeutic for prostate cancer. These viruses are designed to deliver two suicide genes- cytosine deaminase (CD) and herpes simplex type 1 thymidine kinase (HSV-1 TK)- to tumor cells. CD functions to convert 5-fluorocytosine into a toxic 5-FU metabolite, while HSV-1 TK phosphorylates ganciclovir, which converts it into a nucleotide analog and inhibits DNA replication (73). Ad5-CD/TK was administered to prostate cancer patients in a Phase I clinical trial and was deemed safe (74), and an improved Ad5-yCD/mutTK_SR39_-ADP was designed, which contains a more catalytically active mutant herpes TK (SR39) (75). In a phase I trial, nine patients with localized PCa received Ad5-yCD/mutTK_SR39_-ADP in combination with intensity-modulated radiotherapy and no additional toxicities were incurred compared to the first generation Ad5-CD/TKrep. Interestingly, the authors report that patients in the intermediate risk group had improved responses compared to the high-risk group, as 0% of patients within the intermediate-risk group had adenocarcinoma at their last biopsy (24 months) (75). These findings led to the development of new Ad5 therapy that is modified to express human IL-12 (Ad5-yCD/mutTKSR39rep-hIL12), which enhanced T and NK anti-tumor activity in a preclinical mouse model (76). A Phase I trial recruiting patients with locally recurrent prostate cancer for Ad5-yCD/mutTKSR39rep-hIL12 is underway (NCT02555397).

Another oncolytic virus that has been tested in prostate cancer patients in pelareorep (Reolysin), a reovirus. Reovirus oncolysis is activated by the Ras signaling pathway, and can therefore be harnessed as a therapeutic for cancer (77, 78). Reovirus therapy increases the secretion of pro-inflammatory cytokines, increases CD8 T and NK cell homing to tumors, and increases expression of MHC class I on tumor cells to present tumor antigens (79, 80). In a Phase I trial of 33 patients with malignant solid tumors, five PCa patients were treated with reovirus (type 3 Dearing) for a maximum of 5 days every 4 weeks. Overall, administration of the reovirus was safe with minimal severe adverse event (81). A Phase II clinical trial testing the effects of reovirus in combination with docetaxel and prednisone in patients with mCRPC was also completed, although combination of docetaxel and prednisone with pelareorep presented no survival benefit compared to docetaxel and prednisone alone (82). A Phase II clinical trial with pelareorep in combination with pembrolizumab in patients with pancreatic cancer is ongoing, however there are currently no ongoing trials for peloreorep for prostate cancer.

### Combination Immunotherapies

Despite diverse investigations into immunotherapeutic agents for PCa, the results from each agent as a monotherapy have not shown satisfying benefits. To improve clinical outcomes, combinations of these immune agents with each other or hormonal therapy, chemotherapy, radiation, or surgery may be a necessary and optimal approach. Currently, there are several active clinical trials investigating immunotherapeutic combinations.

#### Vaccines and Immune Checkpoint Blockades

Immune checkpoint inhibitors in combination with vaccination can enhance anti-tumor T cell responses. A phase I trial testing ipilimumab in combination with GVAX in 12 chemotherapy naïve mCRPC patients reported that combination therapy led to a PSA decline of ≥50 in 25% of patients, with minimal adverse events (83). Another phase I trial assessed the combination of ipilimumab and PROSTVAC vaccine in 30 mCRPC patients. In 14 of the 24 chemotherapy naïve patients, PSA declines were observed, with 6 patients having a reduction of ≥50% (49). A study in which 25 mCRPC patients were given combination (either concurrent or sequential) therapy of pembrolizumab and a tumor PAP-specific DNA vaccine showed that 11 of 25 patients had increases in PAP-specific CD8 T cells after treatment. Of the 13 patients that received concurrent combination therapy, 8 patients had serum PSA declines (84). Phase II trials are underway to further investigate these combinations and include Sip-T with anti-PD-1 mAb (CT-011) (NCT01420965), Sip-T with anti-CTLA4 (ipilimumab) (NCT01804465), and PROSTVAC with anti-PD1 (nivolumab) (NCT02933255).

#### AST and Immunotherapy

AST is one of the first line therapies after diagnosis with mCRPC. AST has been shown to induce multiple effects on the immune system, such as modulating cancer cell sensitivity to T cells and increasing T cell infiltration into the prostate (85). Clinical trials are now investigating combination AST and immunotherapies. A randomized phase II trial evaluating Sip-T with standard AST showed higher cytokine responses and CD8^+^ T cell activation when Sip-T was administered after AST (86). Several clinical trials testing combinations of immunotherapies and androgen ablation in mCRPC patients are underway, such as enzalutamide with atezolizumab (NCT03016312), pembrolizumab (NCT03753243), or nivolumab and radiation therapy (NCT03543189).

#### Chemotherapy and Immunotherapy

Chemotherapeutic drugs function through disrupting normal cell cycle, leading to either cell cycle arrest or apoptosis of tumor cells. Tumor cells that are undergoing apoptosis can be classified as immunogenic cell death (ICD), which enhances the “visibility” of tumor antigens to the immune system. In the process of ICD, tumor cells can release damage associated molecular patterns (DAMPs), such as HMGB1, ATP, and CRT (87), leading to subsequent activation of resident immune cells. The use of ICI in combination with chemotherapy could enhance anti-tumor immune activity. A variety of chemotherapy combination trials are being performed including: rucaparib and nivolumab (NCT03572478), ipatasertib and atezolizumab (NCT03673787), evofosfamide and ipilimumab (NCT03098160), and cabozantinib with atezolizumab (NCT03170960).

#### Radiotherapy and Immunotherapy

Radiotherapy causes tumor cell death and subsequent release of TAAs that can be processed and presented to CD8^+^ T cells (88, 89). Although not extensively studied in PCa yet, patients with metastatic pancreatic (90) or melanoma (91) have previously been reported to show tumor regression or complete response after combination immunotherapy and radiotherapy to a limited number of tumor sites inducing an abscopal effect. Radiation therapy, such as Radium-233, brachytherapy, and stereotactic ablative body radiation (SABR), are being combined with Sip-T (NCT02463799, NCT01807065, NCT01818986), pembrolizumab (NCT03093428), and nivolumab (NCT03543189) for treating men with various stages of PCa.

## Potential Mechanisms of Resistance of Prostate Cancer to Immunotherapy

To date, clinical trials of immune therapy for PCa patients have focused on metastatic prostate cancer, with specific efforts being made for progressing therapies for patients with castration resistant disease. A dysfunctional immune system in men with mPC may account for unresponsiveness to current immunotherapy. Patients with metastatic prostate cancer have dysfunctional cellular immunity and an increased immune suppressive tumor microenvironment. These compromised immune functions include, but are not limited to, impaired function and renewal of natural killer (NK) cells (92, 93), and impaired expression of CD3ζ in NK and T cells (94), a key signaling molecule for T-cell receptor (TCR) and NK cell activating receptors. Metastatic CRPC patients also have reduced T cell frequency in the circulation (95), although it is not clear whether it is the outcome of AST or tumor-mediated modulation of T cell homeostasis. Men with mPC also have increased suppressive phenotypes of myeloid suppressor cells and regulatory T cells in the circulation, as well as in the tumor microenvironment (96–98). The compromised cellular immunity and highly immune suppressive tumor microenvironment likely explain the reported low infiltration of effective immune cell types (so-called “cold” tumor) in the primary tumor in men with PCa. Whether lack of immune infiltration is due to inability of effector NK and T cells homing to the tumor, or survival in the local tumor microenvironment, is an open question.

Other potential mechanisms of prostate cancer resistance to immunotherapy have been proposed. One is immune tolerance due to the nature of slow disease progression (99, 100). Low mutational tumor burden may contribute to the *de novo* resistance of prostate cancer to immunotherapy (101), although this view is controversial as stratified genomic analyses revealed higher mutational burden than found in renal cancer (102). Taken together, the immunosuppressive prostate TME creates a challenge for developing effective immunotherapeutics. Development of mechanism-driven immunotherapies that can restore NK and CD8 T cell function, as well as overcome prostate immune suppressive tumor microenvironment, are needed for effective treatment of metastatic prostate cancer.

## Promising Preclinical Studies

### Animal Models

One widely-used PCa mouse model is TRAMP (transgenic adenocarcinoma of the mouse prostate), which expresses SV40 T antigen under the rat probesin promoter. Due to SV40 dependent disruption of p53 and pRb function, TRAMP mice spontaneously develop prostate tumors that are detectable at 18–24 weeks of age (103). The advantage of TRAMP mice tumor progression is that it mimics human pathology well, with natural progression from initial hyperplasia, intraepithelial neoplasia, adenocarcinoma, expression of neuroendocrine markers in older mice, as well as metastasis into lung and lymph node tissues (103). TRAMP tumors develop androgen independence early, providing a good model for androgen-independent disease states (104). Another commonly used mouse model is LADY, which also expresses SV40 under a large promoter fragment of the PB gene (LBP) is also commonly used. The timeline to disease is slightly increased compared to the TRAMP models (10–15 weeks to initial hyperplasia) (105). Other mouse models that are used have prostate specific Cre-lox gene deletion of Pten, p53, or Smad4, and prostate-specific overexpression of c-Myc or N-Myc (106). PDX models (CrownBio) of PCa, specifically CRPC, are being developed for their eventual use in humanized mouse systems. Further research will be needed in the development of humanized PDX models of prostate cancer due to the difficulties of propagating prostate tumors *ex vivo* (107).

### Promising Proof-of-Concept New Therapeutic Investigations

#### Targeting Tumor-Released Soluble MIC to Harness NKG2D Pathway

MIC (major histocompatibility I chain-related molecule) is an NKG2D ligand expressed during early stages of cancer on stressed cells. Engagement of NKG2D with membrane bound MIC results in the stimulation of T and NK cells. However, as the cancer progresses, proteases cleave MIC from the surface, producing soluble MIC (sMIC). sMIC contributes to an increased immunosuppressive tumor microenvironment by promoting myeloid-derived suppressor cells (MDSC) expansion (108) and reducing the expression of NKG2D on T and NK cells (93). High serum concentrations of soluble NKG2D ligands are associated with poor prognosis in a variety of cancers including prostate (93), melanoma (109), and hepatitis B induced hepatocellular carcinoma (HCC) (110). PCa patients with metastatic disease have significantly elevated levels of serum sMIC than those with localized disease. Targeting sMIC with a non-blocking anti-MIC antibody can effectively de-bulk prostate tumor burden and eliminate distant metastasis by re-invigorating endogenous innate and adaptive antitumor responses in preclinical models of prostate cancer (108, 111–113). Antibody targeting sMIC also sensitizes mouse prostate tumors to treatment with anti-CTLA4 (111) and PD-1/PD-L1 blockade (114).

#### CLTA-4 and PD-1 Checkpoint Blockade in Combination With MDSC Targeted Therapy

Myeloid derived suppressor cells (MDSCs) are a recently characterized immune cell subset implicated in inflammatory processes and tumor progression (115). MDSCs promote an immunosuppressive TME through a variety of mechanisms including arginase I production (116), IL-10 immunomodulation (117), expression of PD-L1 (118), and others (119) which all contribute to the inhibition of T cell function. Recently it was described that IL-23 secreted by MDSCs drives mCRPC development in mice through androgen receptor activation, and that blockade of IL-23 restored sensitivity to androgen deprivation (120). Other therapies have been investigated to inhibit MDSC functionality. Lu et al. (121) tested combination anti-CTLA4 and anti-PD1 (ICI) with either dasatinib (tyrosine kinase inhibitor), cabozantinib (tyrosine kinase inhibitor), or BEZ235 (phosphoinositide 3-kinase/mTOR dual inhibitor) to target tumor MDSCs in a mouse mCRPC model. They found the combination therapy of ICI + dasatinib and ICI + cabozantinib led to lower tumor mass and fewer metastases compared to ICI alone. Additionally, there were lower percentages of intratumoral granulocytic MDSCs, as well as an increase in intratumoral CD8^+^/Treg ratios (121). These findings show promise for combination ICI and MDSC suppression therapies.

#### Oncolytic Virus-Mediated Immunotherapy

As mentioned earlier, oncolytic viruses are being developed as an immunotherapeutic modality for prostate cancer. Many oncolytic viruses, as a monotherapy, have already completed Phase I/II clinical trials for prostate cancer [reviewed in (69)] with no survival benefit for patients. Preclinical studies are shedding light on how oncolytic viruses impact the immune system to generate anti-tumor responses. Oncolytic viruses have been genetically modified to express human cytokines and other immune modulators, such as bispecific T cell engagers (BiTEs).

In one preclinical model, mice bearing CMR22 prostate tumors were treated with oncolytic herpes simplex virus-1 (oHSV) expressing human IL-12 (NV1042) in combination with vinblastine, a microtube disrupting agent. Combination of NV1042 and vinblastine resulted in reduced tumor growth compared to either treatment alone (122, 123). Also in development in preclinical studies is an oncolytic adenovirus expressing BiTEs. In this study, fresh prostate biopsies were treated with an oncolytic group B adenovirus, enadenotucirev (EnAd), to express a BiTE specific for fibroblast activation protein (found on cancer associated fiborblasts) and CD3ε. Treatment with the EnAd-BiTE led to an increase in T cell activation and cytotoxicity against tumor stromal fibroblasts (124). These are exciting new developments in harnessing the unique feature of oncolytic virus to prime tumor microenvironment for active immune responses.

## Concluding Remarks

In all, there is substantial progress being made in understanding the biology of cancer immunotherapy for prostate cancer. Within the last 10 years there have been a series of results from mono-immunotherapy trials which have resulted in minimal survival enhancement in mCRPC patients, leading to a need to investigate combinatorial therapeutic approaches. The immunosuppressive environment within PCa poses a challenge the development of effective immunotherapies. One general trend in current combinatorial approaches involves checkpoint blockade inhibitors with vaccination (cell, viral, DNA, or peptide based); “hitting the brakes” on immunosuppression, and “pushing the gas pedal” for immune activation.

Other immunosuppressive mechanisms within PCa are being identified through preclinical trials, leading to new therapeutic targets that could be tested in the clinic within the next few years. As new technologies become available, in depth immune cell characterization within mCRPC tumors will be necessary to gain a better understanding of other, currently unidentified, immunosuppressive cell types that may be present. Such cellular characterizations could pave the way for the discovery of new therapeutic drug targets.

Overall, discovery of single agent immunotherapies for prostate and other “cold” cancers has been robust in current years. As results are coming out of these studies, it is clear that combinatorial approaches will be needed to enhance immune responses in prostate cancer patients. There are currently over one hundred immunotherapy based clinical trials that are ongoing for prostate cancer, many of which are testing combination therapies. As we begin to see the results of these ongoing studies, as well as new therapeutics moving into the clinic, we will understand novel ways to increase the potential of immunotherapies for prostate cancer.

## Author Contributions

AB and JW wrote manuscript. AU, AM, DV, JS, and JW edited and revised manuscript.

### Conflict of Interest Statement

JW holds patents of antibody targeting tumor-derived soluble NKG2D ligand sMIC. The remaining authors declare that the research was conducted in the absence of any commercial or financial relationships that could be construed as a potential conflict of interest.
